# Guard-cell-targeted overexpression of Arabidopsis *Hexokinase 1* can improve water use efficiency in field-grown tobacco plants

**DOI:** 10.1093/jxb/erac218

**Published:** 2022-05-20

**Authors:** Liana G Acevedo-Siaca, Katarzyna Głowacka, Steven M Driever, Coralie E Salesse-Smith, Nitsan Lugassi, David Granot, Stephen P Long, Johannes Kromdijk

**Affiliations:** International Maize and Wheat Improvement Center (CIMMYT), El Batan, Mexico; Carl R. Woese Institute for Genomic Biology, University of Illinois at Urbana-Champaign, Urbana, IL, USA; Carl R. Woese Institute for Genomic Biology, University of Illinois at Urbana-Champaign, Urbana, IL, USA; Department of Biochemistry, University of Nebraska-Lincoln, Lincoln, NE, USA; Carl R. Woese Institute for Genomic Biology, University of Illinois at Urbana-Champaign, Urbana, IL, USA; Carl R. Woese Institute for Genomic Biology, University of Illinois at Urbana-Champaign, Urbana, IL, USA; Institute of Plant Sciences, Agricultural Research Organisation, The Volcani Center, Bet Dagan, Israel; Institute of Plant Sciences, Agricultural Research Organisation, The Volcani Center, Bet Dagan, Israel; Carl R. Woese Institute for Genomic Biology, University of Illinois at Urbana-Champaign, Urbana, IL, USA; Lancaster Environment Centre, University of Lancaster, Lancaster, UK; Carl R. Woese Institute for Genomic Biology, University of Illinois at Urbana-Champaign, Urbana, IL, USA; Department of Plant Sciences, University of Cambridge, Cambridge, UK; University of Essex, UK

**Keywords:** CO_2_ assimilation, hexokinase, photosynthesis, water use efficiency

## Abstract

Water deficit currently acts as one of the largest limiting factors for agricultural productivity worldwide. Additionally, limitation by water scarcity is projected to continue in the future with the further onset of effects of global climate change. As a result, it is critical to develop or breed for crops that have increased water use efficiency and that are more capable of coping with water scarce conditions. However, increased intrinsic water use efficiency (iWUE) typically brings a trade-off with CO_2_ assimilation as all gas exchange is mediated by stomata, through which CO_2_ enters the leaf while water vapor exits. Previously, promising results were shown using guard-cell-targeted overexpression of *hexokinase* to increase iWUE without incurring a penalty in photosynthetic rates or biomass production. Here, two homozygous transgenic tobacco (*Nicotiana tabacum*) lines expressing Arabidopsis *Hexokinase 1* (*AtHXK1*) constitutively (35SHXK2 and 35SHXK5) and a line that had guard-cell-targeted overexpression of *AtHXK1* (GCHXK2) were evaluated relative to wild type for traits related to photosynthesis and yield. In this study, iWUE was significantly higher in GCHXK2 compared with wild type without negatively impacting CO_2_ assimilation, although results were dependent upon leaf age and proximity of precipitation event to gas exchange measurement.

## Teaser

Guard-cell-targeted overexpression of Arabidopsis *Hexokinase 1*in tobacco led to decreases in stomatal conductance under mild water-limiting stress, which decreased rates of CO_2_assimilation but enhanced photosynthetic water use efficiency.

## Introduction

Water availability is considered one of the largest limitations to crop productivity in the world and is expected to become more of an issue in the future as water scarcity is predicted to increase due to climate change. Despite the limitation of water availability on agricultural production, crop irrigation accounts for 70% of global freshwater use (FAO, 2017). To improve sustainability in agricultural water management, development of plants with inherently higher water use efficiency could help decrease soil water depletion throughout the growing season and thereby decrease reliance on irrigation (Condon *et al.* 2004). Plants control water loss to the atmosphere via stomata, microscopic pores on the leaf surface (Dong and Bergmann, 2010; Buckley *et al.*, 2020; Lawson and Matthews, 2020) that consist of a pair of guard cells (Dong and Bergmann, 2010; Misra *et al.*, 2015). These stomatal pores form the main gateway for gaseous exchange of the plant shoot with the surrounding atmosphere, controlling both photosynthetic CO_2_ uptake and water loss via transpiration. The aperture of stomatal pores is affected by changes in guard cell turgor pressure (Misra *et al.*, 2015; Lawson and Matthews, 2020). Stomatal movements respond to a wide range of cues, including light intensity, CO_2_ concentration, leaf-to-air vapor pressure deficit and soil water availability via a complex set of intertwined regulatory networks that aim to maintain healthy and sustainable plant growth and development (Daszkowska-Golec and Szarejko, 2013; Bertolino *et al.*, 2019; Grossiord *et al.*, 2020; Lawson and Matthews, 2020; Matthews *et al.*, 2020).

Genetic manipulation of signal perception, signal transduction, or signal translation underlying stomatal movement in response to external cues can be used to adjust the genetic set-point for stomatal control over water loss and carbon gain. Indeed, reductions in transpiration and improvements in water use efficiency have been demonstrated repeatedly through genetic manipulation approaches, such as overexpressing the phytohormone abscisic acid (ABA), which triggers stomatal closure (Thompson *et al.* 2007), overexpression of the thylakoid lumen pH sensor photosystem II subunit S, which reduces light-induced stomatal opening (Głowacka *et al.* 2018), and overexpression of Arabidopsis *Hexokinase 1* (*AtHXK1*), which triggers stomatal closure in response to cellular sugar status (Kelly *et al.* 2013).

Whereas these manipulations give rise to plants with more conservative water use, they often do so at the expense of carbon gain or biomass accumulation under well-watered conditions. However, for *AtHXK1* overexpression, this trade-off may be ameliorated by targeting the overexpression to guard cells. The native hexokinase (HXK) protein is expressed ubiquitously throughout cell types. It possesses hexose phosphorylation activity and is the only enzyme that can phosphorylate glucose in plants (Granot *et al*. 2013, 2014). Aside from its catalytic properties, HXK also mediates source–sink interactions, down-regulating photosynthetic gene expression in response to sugar accumulation in photosynthetic mesophyll cells. Previously, plants that constitutively overexpress *AtHXK1* from Arabidopsis were shown to be inhibited in photosynthesis and growth (Jang *et al.* 1997; Dai *et al.* 1999; Kelly *et al.* 2012). However, *HXK* overexpression targeted to guard cells seems to stimulate stomatal closure in response to sugar accumulation from the transpiration stream (Kelly *et al.* 2013). Guard-cell-specific expression of *AtHXK1* driven by the promoter of the potato guard cell potassium channel gene *KST1* (Műller-Röber *et al.* 1995) led to decreased stomatal conductance and increased water-use efficiency, without adversely affecting photosynthesis and growth as reported for constitutive overexpression (Lugassi *et al.* 2019; Kelly *et al.* 2019).

Guard-cell-targeted expression of *AtHXK1* therefore seems to have the potential to improve water use efficiency, as well as tolerance to drought and salt stress (Lugassi *et al*. 2015, 2019). However, these phenotypes have so far only been evaluated in controlled-environment cabinets or greenhouses, but not under field conditions. To improve our understanding of the potential benefits of guard-cell-targeted *AtHXK1* expression, the current study therefore aimed to assess the effects of overexpression of *AtHXK1* on biomass productivity and leaf-level gas exchange of tobacco under rain-fed and irrigated field conditions, using transgenic tobacco plants overexpressing *AtHXK1* in guard cells from Lugassi *et al.* (2019) as well as two newly created tobacco lines with constitutive *AtHXK1* overexpression.

The results showed that the effect of *AtHXK1* overexpression strongly depends on soil water availability and could be affected by plant age. Under well-watered conditions, only minor genotypic differences were discernible, but under mild water-limitation stress in rain-fed plants, the overexpression of *AtHXK1* resulted in sharp declines in stomatal conductance and increased water use efficiency, especially when targeted to the guard cells. Despite these effects on leaf gas exchange, biomass accumulation was not significantly affected in the plants with guard-cell-targeted overexpression of *AtHXK1*, suggesting that this strategy may indeed have potential to improve water use efficiency without pronounced drawbacks on crop productivity.

## Materials and methods

### Plant transformation

*Nicotiana tabacum* cv. ‘Samsun NN’ plants were grown in a mixture of 70% tuff and 30% peat (Even Ari, Israel) in a temperature-controlled greenhouse. Transformations were performed using *Agrobacterium tumefaciens* strain EHA105 harboring the pGA643 binary vector containing *35Spro::AtHXK1* (*HXK1* locus: *AT4G29130*) as described in Dai *et al.* (1999) and Gallois and Marinho (1995). Transgenic plants were identified using a kanamycin-resistance assay followed by PCR confirmation of the transgene within T_0_ plants. Identification of T_1_ homozygous plants was conducted by testing the presence of the transgene by PCR in 20 T_2_ seedlings that were collected from each individual T_1_ plants. For the PCR, primers matching *35Spro* (forward) and *AtHXK1* (reverse; Supplementary Table S1) were used.

### RNA extraction, cDNA synthesis, and RT quantitative PCR

Leaf tissue was harvested from WT and 35SHXK plants (see below), and total RNA was extracted from the leaf tissue using the LogSpin method (Yaffe *et al.*, 2012). Samples were ground using a Geno/Grinder (SPEX SamplePrep, Metuchen, NJ, USA) and RNA was extracted in 8 M guanidine hydrochloride buffer (Duchefa Biochemie) and transferred to tubes containing 96% ethanol (BioLab, Jerusalem, Israel). Then, samples were transferred through a plasmid DNA extraction column (RBC Bioscience, New Taipei City, Taiwan), followed by two washes in 3 M Na-acetate (BDH Chemicals, Mumbai, India) and two washes in 75% ethanol, and eluted with diethylpyrocarbonate water (Biological Industries Israel Beit Haemek LTD, Kibbutz Beit Haemek, Israel) that had been preheated to 65 °C. The RNA was treated with RQ1-DNase (Promega, Madison, WI, USA) according to the manufacturer’s instructions, to degrade any residual DNA. For the preparation of cDNA, total RNA (1 μg) was taken for reverse transcription–PCR using the qScriptTM cDNA Synthesis Kit (Quanta BioSciences, Gaithersburg, MD, USA) following the manufacturer’s instructions. cDNA samples were diluted 1:5 in double-distilled water. Quantitative real-time PCR reactions were performed using SYBR Green mix (Thermo Fisher Scientific, Waltham, MA, USA) and reactions were run in a RotorGene 6000 cycler (Corbett, Mortlake, NSW, Australia). Following an initial pre-heating step at 95 °C for 15 min, there were 40 cycles of amplification each consisting of 10 s at 95 °C, 15 s at 55 °C, 10 s at 60 °C, and 20 s at 72 °C. Results were analysed using the RotorGene software. The expression levels of *AtHXK1* were normalized using tobacco actin (XM_016658252) as a reference gene (Supplementary Table S2) and relative expression levels were calculated using ΔΔ*C*_t_.

### Plant propagation for field experiment

For the field experiment, we used two homozygous transgenic *Nicotiana tabacum* cv ‘Samsun NN’ lines expressing *AtHXK1* constitutively (35SHXK2 and 35SHXK5), as well as line GCHXK2 from Lugassi *et al.* (2019), which has guard-cell-targeted overexpression of *AtHXK1* under the *Solanum tuberosum KST1* partial promoter, and wild-type (WT) control. The two 35SHXK lines differed in *AtHXK1* transcript levels, with 35SHXK5 showing approximately 4-fold higher transcript levels than 35SHXK2 (see Supplementary Fig. S2).

Seeds were germinated on growing medium (LC1 Sunshine mix, Sun Gro Horticulture, Agawam, MA, USA) in the greenhouse on 7 May 2018 and 3 June 2020. Five days after germination, seedlings were transplanted to floating trays (Transplant Tray GP009 6 × 12 cells, Speedling Inc., Ruskin, FL, USA), filled with specialized growing medium for hydroponics (Coco Loco potting mix, Fox Farm, Arcata, CA, USA) and grown hydroponically in solution-filled tubs. The concentration of dissolved nutrients in solution was checked every 2 d with a hand-held total dissolved solids meter (COM-100, HM Digital Inc., Culver City, CA, USA) and adjusted to 150 ppm by addition of 20–10–20 water-soluble fertilizer (Jack’s Professional, JR Peters Inc., Allentown, PA, USA) when required. Five days after the transplant to trays, etridiazole fungicide (Terramaster 4EC to a final concentration of 78 µl l^−1^, Compton Manufacturing Co. Inc., Middlebury, CT, USA) was added to the solution to protect the plants against root fungus disease in the field. Two applications of mancozeb (Dithane Rainshield Fungicide at 1 g l^−1^, Dow AgroSciences Canada Inc., Calgary, Alberta, Canada) were applied 10 and 15 d after transplant to prevent foliar fungus disease.

### Field experiment

The field experiment took place over the course of two field seasons, one in May–July 2018 and the other in June–August 2020. The same ­experimental design and genotypes were used between years. Seedlings were transplanted to an experimental field site at the University of Illinois Energy Farm (40.11°N, 88.21°W, Urbana, IL, USA) on 31 May 2018 and 29 June 2020. The field was prepared 1.5 weeks before transplant by rototilling, cultivation, and harrowing. At this time chlorpyrifos (1.5 g m^−2^ Lorsban 15G Insecticide, Dow AgroSCiences Canada Inc.) was worked into the soil to suppress cutworm damage, sulfentrazone (29 µl m^−2^ Spartan 4F pre-emergence herbicide, FMC Agricultural Solutions, Philadelphia, PA, USA) was applied to reduce the emergence of weeds, and slow-release fertilizer (30.8 g m^−2^ ESN Smart Nitrogen, Agrium US Inc., Denver, CO, USA) was put down. After transplant, all seedlings were sprayed with thiamethoxam (7 mg per plant, Platinum 75 SG insecticide, Syngenta Crop Protection LLC, Greensboro, NC, USA) to prevent damage from insect herbivory. Fermentation solids, spores, and insecticidal toxins from *Bacillus thuringiensis* subsp. *Kurstaki* strain ABTS-351 (2.6 ml l^−1^, DiPel Pro dry flowable biological insecticide, Valent Biosciences Corp.) were applied once a week to suppress tobacco hornworm.

The experimental design consisted of two sets of six randomized complete blocks of 6 × 6 plants, spaced 38 cm apart. The central 16 plants of each block were divided into four rows of four plants per genotype in the north–south direction, surrounded by one border row of WT. Per set the blocks were positioned in a 2 (N–S) by 3 (E–W) rectangle, with 75 cm spacing between blocks. The two sets were positioned in the N–S direction and separated by an empty strip of 2.5 m. The entire experiment was surrounded by two rows of wild-type. Each set was subjected to a different irrigation treatment. In both years, the set at the north side of the experiment was used for the rain-fed treatment, where water availability was exclusively determined by rain events during the experiment. The set at the south side of the experiment was used for the irrigated treatment, where watering was provided through parallel drip irrigation lines (17 mm PC Drip Line no. DL077, The Drip Store, CA, USA). The irrigation was used daily to return the soil to field capacity 1 h before sunset, except for days during or 1 d after a rain event. The soil in this field site is classified as silt loam/silty clay loam, which can be relatively poorly draining. To improve draining after watering and precipitation events, trenches with a depth of approximately 10 cm were dug between the blocks of each set and connected to 15 cm-deep drains that flanked the experiment with 3 m spacing on both east and west sides. Excess water could be released to a neighboring field by sump-pumps positioned in each drain. Soil water content was monitored by soil moisture probes (EC-5, METER Group Inc., Pullman, WA, USA) inserted following the manufacturer’s recommendations at a depth of 15 cm at the center of each block, as well as measured twice a week around mid-day with a handheld soil moisture sensor (HydroSense II with CS658 sensor mounted on an insertion pole, Campbell Scientific, Logan, UT, USA) to sample the soil moisture content of the upper soil profile at five different positions in each block. Light intensity (LI-190R quantum sensor, LI-COR, Lincoln, NE, USA) and air temperature (Model 109 temperature probe, Campbell Scientific) were measured directly adjacent to the experiment. Half-hourly averages of climate and soil water content were logged using a data-logger (CR1000, Campbell Scientific) except for a period of two weeks in early July 2020 when the sensor data was not logged due to a technical issue. Precipitation was measured close to the field using a precipitation gauge (NOAH IV Precipitation Gauge, ETI Instrument Systems Inc., Fort Collins, CO, USA).

The number of leaves per plant was determined for all plants twice a week throughout the experiment, as well as the visible presence of the terminal flower bud on the main stem (towards the end of the experiment). The experiment was terminated by a final harvest on 11 July 2018 and on 17 August 2020. At final harvest, stem length and number of leaves per plant were determined destructively and total leaf area per plant was measured with a conveyor-belt scanner (LI-3100C Area Meter, LI-COR). Leaf, stem, and root fractions were dried to constant weight at 60 °C in a custom-built drying oven, after which dry weights were determined.

### Photosynthetic gas exchange: diurnal and CO_2_ response curve measurements

Diurnal gas exchange measurements were performed on 15 June and 30 June 2018, and 8 July, 23 July, 28 July, and 12 August 2020. A total of six diurnal gas exchange measurements were made across both years. Diurnal photosynthetic gas exchange was determined from 07.00 to 19.00 h at 2 h intervals using an open gas exchange system (LI6400XT (2018) and LI6800 (2020), LI-COR) with integrated leaf chamber fluorometer. On 30 June 2018, early morning measurements had to be delayed until 09.00 h to allow all morning dew to evaporate. On 23 July 2020, two afternoon measurements were skipped due to a nearby storm and inclement weather. At each time point, ambient light intensity was first measured using the external PAR-sensor of the infra-red gas analyser. Light intensity in the cuvette was then set to equal the measured ambient intensity (using 90% red and 10% blue), block temperature was set to measured air temperature, and CO_2_ concentration in the airstream was set to 400 µmol mol^−1^. The youngest fully expanded leaf of one randomly selected plant per genotype per block was clamped in the cuvette and gas exchange values were logged as soon as stomatal conductance reached steady rates for 10 s (based on visual assessment), which happened typically after 1.5–2 min, but could take up to 3–4 min on some occasions.

Photosynthetic capacity was determined from CO_2_ response curves on 29 June 2018. The capacity of photosynthetic biochemistry was determined from CO_2_ response curves of net assimilation rate for all plants from one randomly selected block per irrigation treatment. Leaves were clamped in the gas exchange cuvette with light intensity set to 2000 µmol m^−2^ s^−1^ photon flux density, block temperature of 32 °C and CO_2_ concentration in the airstream set to 400 µmol mol^−1^. When steady state rates of CO_2_ exchange and stomatal conductance were reached, CO_2_ concentration was changed from 400 to 300, 200, 150, 100, 75, 400, 400, 600, 900, 1200, and 1500 µmol mol^−1^, and gas exchange parameters were logged when the coefficient of variation for net assimilation rate and intercellular CO_2_ concentration (*C*_i_) averaged over 10 s became less than 1% (waiting time between 3 and 5 min). Ribulose bisphosphate carboxylation capacity (*V*_cmax_), regeneration capacity at 2000 µmol m^−2^ s^−1^ (*J*_2000_), and triose phosphate utilization capacity (*V*_TPU_) were estimated by fitting the biochemical model for leaf photosynthesis (Farquhar *et al.* 1980) and temperature corrections according to Bernacchi *et al*. (2002, 2003).

### Stomatal microscopy

Samples for stomatal microscopy were taken on 7 July 2018. Fresh leaf samples were taken from the youngest fully expanded leaf of field-grown 35SHXK2, 35SHXK5, GCHXK2, and WT plants and mounted onto a microscope slide using double-sided tape. An optical topometer (µsurf explorer, Nanofocus, Oberhausen, Germany) was used to determine stomatal density and size characteristics of the stomatal complex. For stomatal density, a ×20/0.60 objective lens was used to image three randomly selected leaf area fields of view of 0.8 × 0.8 mm^2^ (i.e. total area of 1.92 mm^2^) for each leaf side for four biological replicates per genotype by irrigation treatment combination. To determine the dimensions of stomatal complexes, a ×50/0.80 objective lens was used to image a randomly selected leaf area segment of 0.32 × 0.32 mm^2^ for each of four biological replicates per genotype by irrigation treatment combination. Stomatal complex dimensions (length, width, and area) were estimated from all stomatal complexes that were fully visible within each segment, providing an average of *n*=36 stomatal complexes analysed per group mean.

### Statistical analysis

Statistical analyses were performed using SAS (SAS Studio v3.8, SAS Institute Inc., Cary, NC, USA) and R (R Foundation for Statistical Computing, Vienna, Austria). A generalized linear mixed model (PROC GLIMMIX) with irrigation treatment and date and two-way interaction as the fixed effects and plot as random effect was used for analysis of variance in the discrete soil moisture content measurements. For final harvest and photosynthetic capacity datasets, fixed effects were modelled for irrigation treatment and genotype nested within irrigation treatment and plot as a random effect. For diurnal gas exchange data, two-way interactions were included as fixed effects, while accounting for repeated measures at each time point. For stomatal microscopy data, leaf-side (abaxial or adaxial) and two-way interactions with irrigation treatment and genotype were included as fixed effects, and leaf sample was included as a random effect for the stomatal pore size data. Covariance structures were modelled using the Huynh–Feldt correction. In all cases, normal distribution of modelled residuals was verified using the Shapiro–Wilk test or visual inspection (for the larger datasets). Significant fixed effects (α=0.05) were followed up by testing of differences between means using the Tukey–Kramer multiple comparison correction.

The decision tree model was developed using the ‘rpart’ and ‘rpart.plot’ packages in R and utilized the data from the diurnal gas exchange measurements. Data were randomized and divided into training and test sets (80:20). The model was based on the likelihood of having a significant difference between genotypes for intrinsic water use efficiency and to then understand which factors played a role in the significant difference. To better understand the effect of plant age and precipitation, these topical data were included in the model as well. For plant age, plants that had fewer than 20 leaves and were not flowering were considered ‘young’ while plants with 20 or more leaves and/or flowering were considered ‘mature’. For precipitation, less than 12 mm of precipitation up to 3 d before diurnal measurements was considered ‘low’, while 12 mm or more of precipitation was considered ‘high’. These thresholds were determined by looking at historical precipitation data in Urbana, IL for the months of June, July, and August (Illinois State Water Survey, 2021) (Supplementary Table S2). The decision tree model had a 91.8% likelihood of accurately categorizing the data.

## Results

### Soil water content

**Fig. 1.**
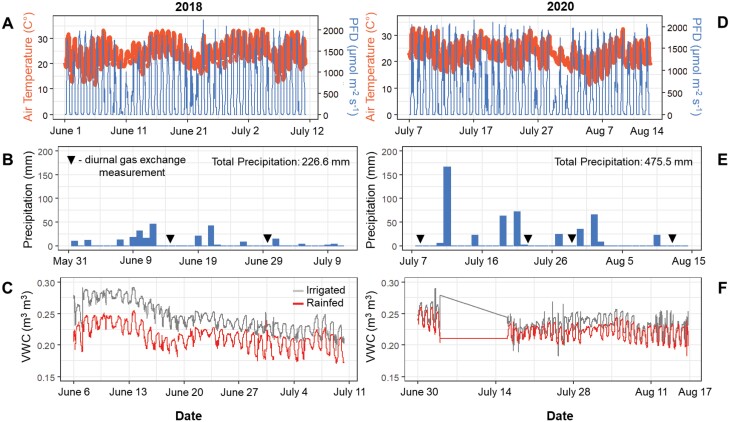
Weather data for 2018 and 2020. (A, B) Air temperature (red) and photon flux density (PFD) (blue) over the course of each growing season. (C, D) Precipitation data for 2018 and 2020. Black triangles represent when diurnal measurements were made in the field. (E, F) Volumetric water content (VWC) measured throughout the growing seasons in both 2018 and 2020. VWC was measured for both the irrigated (gray) and rain-fed (red) treatments.

Two homozygous transgenic *Nicotiana tabacum* cv ‘Samsun NN’ lines expressing *AtHXK1* constitutively (35SHXK2 and 35SHXK5), differing approximately 4-fold in expression of *AtHXK1* transgene (35SHXK5>35SHXK2, Supplementary Fig. S1); line GCHXK2 from (Lugassi *et al.* 2019), with guard-cell-targeted overexpression of *AtHXK1* under the *Solanum tuberosum KST1* partial promoter; and wild-type control plants were grown under irrigated and rain-fed field conditions in a split-plot design. Light intensity and air temperature during the experiment were relatively bright and hot, regularly exceeding 2000 µmol m^−2^ s^−1^ and 30 °C (Fig. 1A, 1D). Precipitation (Fig. 1B, E) mostly occurred via relatively short but strong rainstorm events, and ditches surrounding the plots were effective to rapidly drain water from the topsoil layer via run-off and surface drainage. Based on continuous monitoring using sensors installed at 15 cm depth, the irrigation to field-capacity on days without precipitation combined with the hot, bright, and dry conditions in between precipitation events was sufficient to establish a continuous significant difference in soil water content between the irrigated and rain-fed plots, ranging between 0.02 m^3^/m^3^ and 0.05 m^3^/m^3^ lower soil water content in the rain-fed plots (Fig. 1E) in 2018. Independent probing of the water content in the upper 15 cm soil profile at mid-day across all plots also showed that soil water content was significantly lower in the rain-fed plots by an average of 0.05 m^3^/m^3^ in 2018, except for days immediately following strong precipitation events (Fig. 1C, E). However, 2020 was a considerably wetter year and received more than twice the precipitation observed in 2018 (Fig. 1C, D) resulting in lesser differences between rain-fed and irrigated plots (Fig. 1F). However, significant differences were seen throughout the 2020 and 2018 season for soil water content (Supplementary Fig. S2).

### Biomass productivity and allocation

**Fig. 2.**
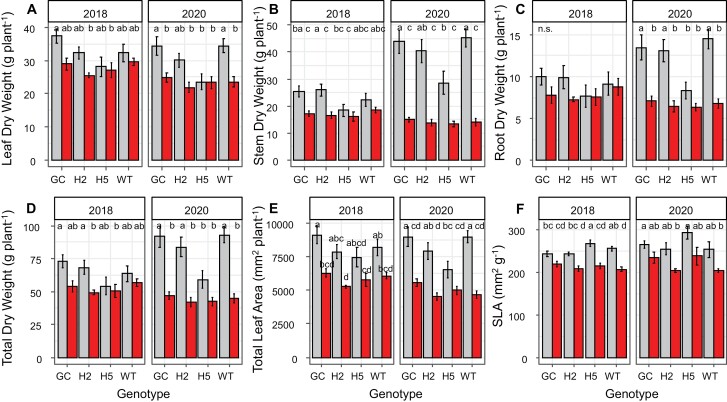
Visualization of biomass and harvest-related traits in 2018 and 2020 including total leaf dry weight (A), total stem dry weight (B), total root dry weight (C), total dry weight (leaves, stems, and roots) (D), total leaf area (E), and specific leaf area (F). Values for irrigated plots are in grey and rain-fed values are represented in red. *n*=6 per genotype and treatment, and error bars depict the standard error for each genotype. Letters are indicative of significant differences between genotypes and treatments where an α<0.05 was applied. Abbreviations of genotypes are as follow: GC, GCHXK2; H2, 35SHXK2; H5, 35SHXK5; WT, wild type.

Leaf area (Fig. 2E), dry-weight accumulation in leaves (Fig. 2A) and stems (Fig. 2B), and total dry-weight per plant (Fig. 2D) were significantly lower in the rain-fed plots relative to the irrigated plots (*P*<0.05) in both 2018 and 2020. Biomass production and leaf counts in 2020 were affected by a severe hailstorm on 11 July that resulted in damage to many of the plants. Consequently, many plants from this field season had axillary growth resulting in some secondary or tertiary stem formation leading to more pronounced differences in stem weight in 2020 (Fig. 2B). In 2018 no significant difference was seen between treatments or genotypes for dry root weight (Fig. 2C). However, root weight was significantly lower (*P*<0.05) in the rain-fed plots in 2020 (Fig. 2C).

Genotype effects were more subtle than the effects of irrigation treatment but were relatively consistent across years (Fig. 2). Under irrigated conditions, the 35SHXK5 plants, which showed the strongest upregulation of hexokinase expression, showed reduced biomass accumulation compared with the other genotypes, whereas the guard-cell-specific overexpression of *HXK1* in GCHXK2 seemed to result in a slight increase in plant size. No consistent trends were detected in leaf appearance rate between years (Supplementary Fig. S3). However, significant differences were identified between seedlings for biomass and leaf counts in both 2018 and 2020 (Supplementary Figs S4, S5).

Leaf area per leaf dry weight (SLA) was significantly higher under irrigated compared with rain-fed conditions (*P*=0.0003) and was significantly affected by genotype (*P*=0.001, Fig. 2F). Irrigated 35SHXK5 plants showed significantly higher SLA than the other genotypes (*P*<0.05) in both 2018 and 2020. The same pattern was also observed for leaf area ratio (LAR), i.e. the leaf area per total dry weight, with significant effects of irrigation treatment and genotype (Supplementary Fig. S6). Under rain-fed conditions, plants from all genotypes developed significantly lower leaf area per total dry weight, relative to the irrigated plots (Supplementary Fig. S6). Within the irrigated plots, 35SHXK5 plants showed significantly higher LAR than the other genotypes (Supplementary Fig. S6).

### Leaf gas exchange: diurnal measurements

**Fig. 3.**
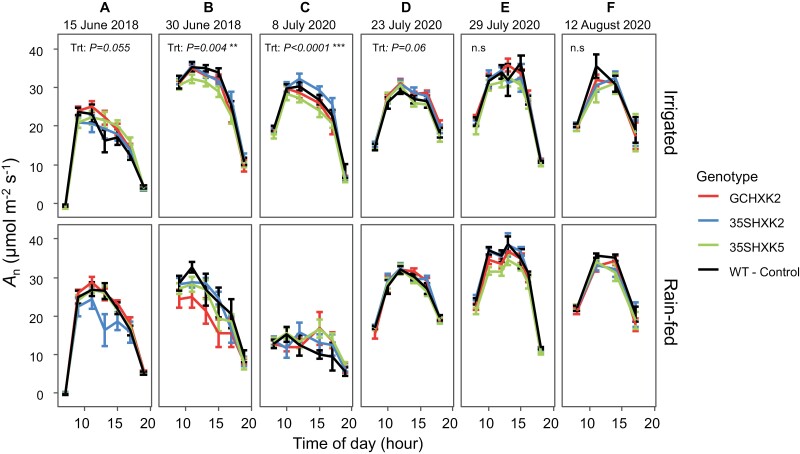
Diurnal data for CO_2_ assimilation (*A*_n_) at six measurement dates during the 2018 and 2020 growing seasons. Diurnal data for *A*_n_ are divided by treatment: irrigated and rain-fed. Measurements on 15 June 2018, and 8 July and 23 July 2020 were early in plant development, as determined by leaf count. Measurements on 30 June 2018, 29 July 2020, and 12 August 2020 were made later in development, as determined by leaf count. Each point of the diurnal curve is the mean of six individuals of each genotype per treatment (*n*=6); error bars represent standard error. The significance threshold is α<0.05; n.s., not significant. ‘Trt’ refers to treatment effect of rain-fed versus irrigated.

No significant difference was found between genotypes throughout the diurnal measurements in both 2018 and 2020 for net CO_2_ assimilation (*A*_n_) (Fig. 3). Despite no significant differences between genotypes, a significant treatment effect was detected on most days that were sampled (*P*<0.05; Fig. 3), demonstrating that the small difference in volumetric soil water content (Fig. 1E, F) were sufficient to affect leaf gas exchange. The values for *A*_n_ were 7.6% higher in irrigated plots than rain-fed plots when compared across both years, all measurements, and all genotypes (Fig. 3). When separated by year, *A*_n_ was 8.3% and 7% greater in irrigated plots in 2018 and 2020, respectively, relative to rain-fed plots (Fig. 3). Additionally, *A*_n_ was overall higher in 2020 relative to 2018 in both the irrigated and rain-fed treatments (Fig. 3).

**Fig. 4.**
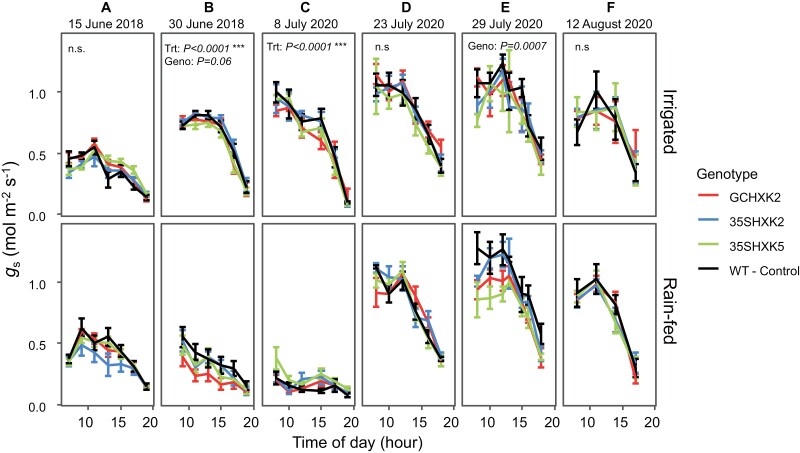
Diurnal data for stomatal conductance of water (*g*_s_) at six measurement dates during the 2018 and 2020 growing seasons. Diurnal data for *g*_s_ are divided by treatment: irrigated and rain-fed. Measurements on 15 June 2018, and 8 July and 23 July 2020 were early in plant development, as determined by leaf count. Measurements on 30 June 2018, 29 July 2020, and 12 August 2020 were made later in development, as determined by leaf count. Each point of the diurnal curve is the mean of six individuals of each genotype per treatment (*n*=6); error bars represent standard error. Significance threshold is α<0.05; n.s., not significant. ‘Trt’ refers to treatment effect of rain-fed versus irrigated, while ‘Geno’ refers to genotype effect (GCHXK2, 35SHXK2, 35SHXK5, and WT) regardless of treatment.

Significant differences were seen between genotypes for stomatal conductance (*g*_s_) in both 2018 and 2020 at later development stages (20-leaf stage) (Fig. 4B, E). In both 2018 and 2020, GCHXK2 had significantly lower *g*_s_ than WT (Fig. 4). GCHXK2 *g*_s_ was 8% and 4% lower than WT in 2018 and 2020, respectively. However, no significant differences were found between genotypes for *g*_s_ earlier during plant development (~10-leaf stage) (Fig. 4). The occurrence of significant treatment effects (*P*<0.05) coincided with diurnals that were performed more than 3 d after a precipitation event, when the upper soil profile had seen sufficient dry-down (Figs 1B, E, 4B, C).

**Fig. 5.**
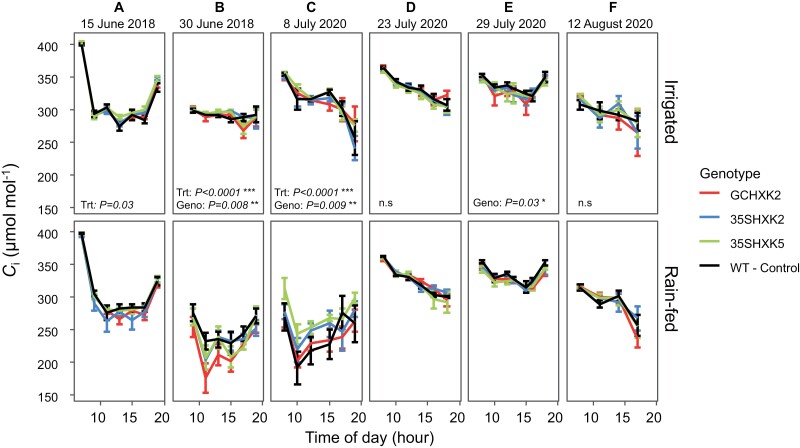
Diurnal data for intercellular CO_2_ concentration (*C*_i_) at six measurement dates during the 2018 and 2020 growing seasons. Diurnal data for *C*_i_ are divided by treatment: irrigated and rain-fed. Measurements on 15 June 2018, and 8 July and 23 July 2020 were early in plant development, as determined by leaf count. Measurements on 30 June 2018, 29 July 2020, and 12 August 2020 were made later in development, as determined by leaf count. Each point of the diurnal curve is the mean of six individuals of each genotype per treatment (*n*=6); error bars represent standard error. Significance threshold is α<0.05; n.s., not significant. ‘Trt’ refers to treatment effect of rain-fed versus irrigated, while ‘Geno’ refers to genotype effect (GCHXK2, 35SHXK2, 35SHXK5, and WT) regardless of treatment.

In 2018 significant differences between genotypes (*P*=0.008) for intercellular CO_2_ concentration (*C*_i_) were only seen later in development at the ~20-leaf stage (Fig. 5B). During this measurement GCHXK2 under rain-fed conditions had the lowest *C*_i_ of all genotypes in both treatments (Fig. 5), suggesting that these plants were enhancing CO_2_ limitation for photosynthesis in favor of lower water loss. Furthermore, the difference in *C*_i_ between GCHXK2 and WT was predominantly found in rain-fed conditions, which coincides with its significantly lower *g*_s_ (*P*=0.0007) on this date (Figs 4, 5). In 2020 significant differences between genotypes were detected at both early and later development stages (*P*=0.009 and *P*=0.03, respectively; Fig. 5C, E). Significant treatment effects for *C*_i_ (*P*=0.03–0.009) mostly coincided with days in which diurnals were measured three or more days after a heavy precipitation event (Figs 1B, E, 5A–C). However, on these dates mean *C*_i_ levels did not differ between WT and GCHXK2, both being very similar across 2020.

**Fig. 6.**
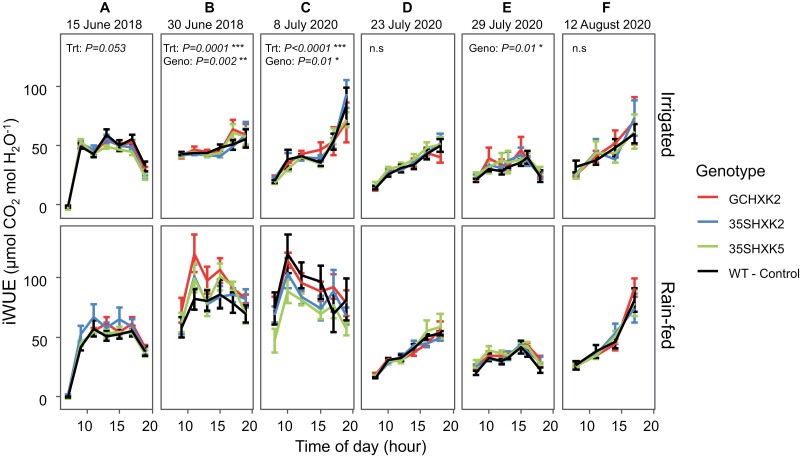
Diurnal data for intrinsic water use efficiency (iWUE*=A*_n_*/g*_s_) at six measurement dates during the 2018 and 2020 growing seasons. Diurnal data for iWUE are divided by treatment: irrigated and rain-fed. Measurements on 15 June 2018, and 8 July and 23 July 2020 were early in plant development, as determined by leaf count. Measurements on 30 June 2018, 29 July 2020, and 12 August 2020 were made later in development, as determined by leaf count. Each point of the diurnal curve is the mean of six individuals of each genotype per treatment (*n*=6); error bars represent standard error. Significance threshold is α<0.05; n.s., not significant. ‘Trt’ refers to treatment effect of rain-fed versus irrigated, while ‘Geno’ refers to genotype effect (GCHXK2, 35SHXK2, 35SHXK5, and WT) regardless of treatment.

Significant differences for iWUE were seen between genotypes in both years as well as at early and late developmental stages (*P*=0.01–0.002; Fig. 6B, C, E). In 2018 GCHXK2 had significantly higher (*P*<0.05) iWUE relative to WT and other transformants under rain-fed conditions (Supplementary Table S2). In 2020, GCHXK2 performed significantly better than the 35SHXK5 and 35SHXK2 transformants and equally well to WT under rain-fed conditions at early development (~10-leaf stage, 8 July 2020) (Fig. 6C). At later development (~20-leaf stage), GCHXK2 had significantly higher iWUE than WT (Supplementary Table S2; Fig. 6E). A significant treatment effect (*P*<0.05) was only seen on two of the dates, both of which were three or more days after a significant rain event (Figs. 1B, E, 6B, C).

### Leaf gas exchange: CO_2_ response curves

CO_2_ response curves of *A*_n_ were measured at the 20-leaf stage and analysed with a model to derive *in vivo* biochemical capacities for CO_2_ assimilation (Supplementary Fig. S7A, B) to assess if the differences in diurnal *A*_n_ could be explained by variation in photosynthetic capacity. However, no significant effects of irrigation treatment or genotype were found for the maximum rate of ribulose bisphosphate (RuBP) carboxylation (*V*_cmax_, Supplementary Fig. S7C), the RuBP regeneration rate at 2000 µmol m^−2^ s^−1^ (*J*_2000_, Supplementary Fig. S7D) and the maximum triose phosphate utilization rate (*V*_TPU_, Supplementary Fig. S7E), demonstrating that the differences in diurnal *A*_n_ did not result from differences in photosynthetic capacity.

### Stomatal density and size

Optical topometry was used in 2018 to examine if the field-grown plants showed any genotype-dependent differences in stomatal density or morphology. Stomatal density on the abaxial leaf side was significantly higher (*P*<0.0001, Supplementary Fig. S8A) compared with the adaxial leaf side, but no significant effects of irrigation treatment or genotype could be detected. The irrigation treatment did affect the geometry of individual pore complexes. Rain-fed plants showed significantly reduced stomatal length and width compared with irrigated plants (*P*=0.0004 and *P*=0.0006, for length and width, respectively, Supplementary Fig. S8B, C). Similarly, the projected surface area per complex (Supplementary Fig. S8D) also varied significantly with irrigation treatment (higher surface area in the irrigated plants, *P*=0.0008). However, neither stomatal density nor size differed significantly between genotypes, suggesting that any observed differences must have arisen from changes in stomatal aperture affecting diffusional limitations to photosynthetic CO_2_ assimilation and transpiratory water release.

### Decision tree model predictions

**Fig. 7.**
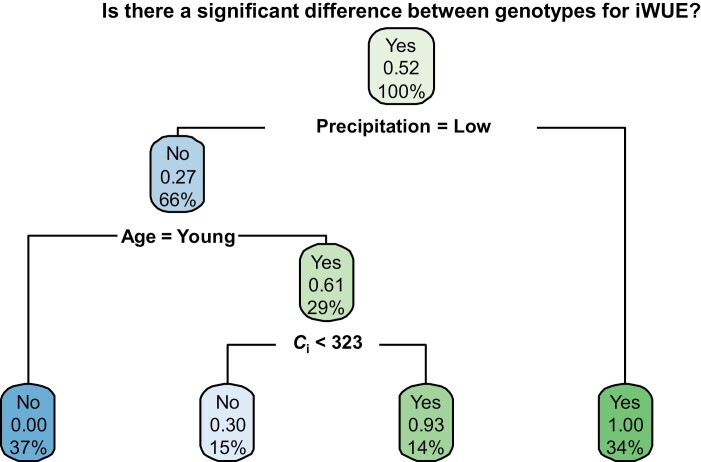
Decision tree model developed to predict the likelihood that a significant difference would be detected between genotypes for intrinsic water use efficiency (iWUE=*A*_n_*/g*_s_) based on factors related to temporal proximity of diurnal measurement to a heavy precipitation event and the age of the plant during the measurement. The number between 0.00 and 1.00 in each node represents the probability that a significant genotype effect for iWUE will be observed, while the percentage in each node shows the relative proportion of measurements that are represented by this node.

To better understand the relationship between precipitation events, plant age, and diurnal gas exchange measurements, a decision tree model was developed to predict factors influencing significant differences (*P*<0.05) between genotypes for iWUE (Fig. 7). The model combined all the diurnal gas exchange data points (~1700 discrete data points) with topical data of plant development (based on leaf count) and temporal proximity to a recent rainfall event to predict the probability of detecting a significant genotype effect on iWUE. Plant age was classified as ‘young’ or ‘mature’ depending on number of leaves and the presence of flowering. Precipitation was classified as ‘low’ and ‘high’, with thresholds developed by looking at historic rainfall during the months of June, July, and August in Urbana, IL (Illinois State Water Survey, 2021).

The resulting model was able to classify data points accurately 91.8% of the time. Across all the gas exchange data there was a 52% likelihood of a significant difference (*P*<0.05) existing among genotypes (Fig. 7). Precipitation played a large role in whether a significant genotype effect on iWUE was likely to be observed. Low precipitation (representing 34% of all data) essentially guaranteed a significant genotype effect on iWUE (Fig. 7). In contrast, for 66% of the data measured in close proximity to a rainfall event, the probability of detecting a difference depended strongly on the plant development stage, with younger plants being more likely to show a significant effect (probability of detecting a significant genotype effect on iWUE *P*=0.61) than mature, flowering plants (*P*=0.00). Finally, for the young plants measured close to a precipitation event, *C*_i_ values below 323 µmol mol^−1^ also correlated with a higher likelihood (*P*=0.93 versus *P*=0.30) of detecting a significant genotype effect for iWUE (Fig. 7).

## Discussion

Often, the improvement of iWUE results in a penalty to *A*_n_ due to tradeoff between the exchange of CO_2_ and water vapor across stomata. However, overexpression of *AtHXK1* under certain conditions could be a promising avenue to improve iWUE without incurring a substantial penalty to *A*_n_ and biomass productivity-related traits. While overexpression of this gene needs to be further examined in the future to determine the conditions under which it would be most helpful, the presented data and previous studies suggest that overexpression of *AtHXK1* may be particularly helpful under water-limiting conditions. Additionally, in situations where there is plentiful water from irrigation or rain-fed conditions, *AtHXK1* ­overexpression may help conserve iWUE in younger plants. Furthermore, this study found that overexpression of *AtHXK1*, both constitutively and in guard cells, did not significantly alter photosynthetic biochemical limitations or stomatal anatomy between genotypes.

### Plant age and proximity of precipitation event play roles in transformant iWUE performance relative to WT

Field-grown tobacco has a moderate water demand relative to other field-grown crops (http://www.fao.org/land-water/databases-and-software/crop-information/tobacco/en/), which progressively increases from the early vegetative growth phase to the rapid growth and elongation phase, before tapering off during flowering and ripening phases (Jones *et al.* 1960). Growth reduction is the easiest observable effect of water deficit. Here, the lack of irrigation in the rain-fed plots was sufficient to constrain growth resulting in reduced dry weight accumulation per plant (Fig. 2B–F), demonstrating that the lack of irrigation in the rain-fed plots was sufficient to constrain growth. Prolonged drought stress in tobacco can also lead to decreased leaf appearance rate (Clough and Milthorpe, 1975) and slower progression through to flowering. These symptoms were not clearly observed in the 2018 field season, suggesting that the levels of stress resulting from the water deficit between the irrigated and rain-fed plots were relatively mild. Symptoms were more pronounced in 2020 where flowering was delayed with rain-fed treatment (Supplementary Fig. S9). This may have been the result of a stronger water limitation at the beginning of the 2020 field season (Supplementary Fig. S2).

The magnitude of water limitation played a central role in gas exchange performance between genotypes, particularly regarding iWUE. We applied decision tree model analysis to help understand the conditions under which a significant difference (*P*<0.05) for iWUE between the genotypes was most likely to be observed (Fig. 7). Significant differences were most likely to be seen between genotypes during periods of low precipitation. Under these conditions of lower precipitation, older plants (~20-leaf stage) were more likely to benefit from guard cell specific overexpression of *AtHXK1*. In some cases, GCHXK2 performed significantly better (*P*<0.05) than WT for iWUE (such as on 30 June 2018 and 29 July 2020) and they never performed significantly worse than WT (Supplementary Tables S2–S5). These findings are consistent with the hypothesis that guard-cell-specific overexpression of *AtHXK1* could help conserve iWUE performance in plants at later stages in development. If so, this could be particularly helpful as older plants—especially as they move towards their reproductive stages—can become more susceptible to yield losses from drought stress (Farooq *et al.* 2009). During vegetative stages, drought stress can result in an eventual yield loss of 25–60%, but this yield loss can increase up to 94% once the plant has reached the reproductive or grain filling stage (Farooq *et al.* 2009).

Conversely, in cases where there was high precipitation it was more likely to see a significant difference between genotypes if the plants were younger (~10-leaf stage). This could lead to an important application in the future, as seedlings or younger plants that experience water stress may be more prone to stunting, which can affect performance throughout the entire growth season. Additionally, seedlings and younger plants are particularly vulnerable to drought stress as their root systems are not as developed and may not be able to access moisture deeper in the soil (Westerband *et al.* 2019). Increasing iWUE in young plants or seedlings, even under non-stress conditions, could help preserve soil water content, which could help buffer the plants from future water stress and its negative impacts on productivity. Additionally, these results suggest that *HXK1* expression in the guard cell could still be beneficial even under conditions in which strong water stress is not present. On days where a significant genotype effect (*P*<0.05) was found for iWUE, the rain-fed GCHXK2 line was consistently in the highest water use efficiency group (Fig. 6; Supplementary Table S2).

Lugassi *et al.* (2019) showed that under controlled conditions, the GCHXK2 transformant performed consistently better than WT. Under field conditions, our results relating to iWUE suggest that these benefits could be highly dependent upon recent precipitation events and plant age, although overexpression of *AtHXK1* targeted to guard cells could still be an avenue to improve iWUE while conserving *A*_n_ performance.

### Impact of no irrigation across the measured traits

The potential of using *AtHXK1* to improve water use efficiency under water-limiting conditions seems to depend strongly on the magnitude and timing of water limitation. Here, it appears that the lack of irrigation manifested itself in two main ways in the plants: (i) the treatment was strong enough to produce a consistent and significant effect (*P*<0.05) on physiological traits such as stomatal size and biomass-related traits (Supplementary Fig. S8; Fig. 2), while (ii) being potentially insufficient to consistently affect traits related to photosynthesis and its biochemical limitations (Figs 3–6).

The treatment was not strong enough to significantly affect stomatal density, which has been shown to decrease under drought stress in rice, wheat, wild rice species, and *Populus* to limit water loss (Hamanishi *et al.* 2012; Ouyang *et al.* 2017). However, rain-fed plants had significantly lower stomatal width and height (*P*=0.0006 and *P*=0.0004, respectively). Previously, it was shown that smaller stomata can help increase water use efficiency in plants as they are able to open and close more quickly relative to larger stomata (Drake *et al.* 2013). Having smaller stomata may then be beneficial under water-limiting conditions as they allow the plant to respond more quickly to stress, such as water limitation, or favorable conditions, such as when water is plentiful after a precipitation event. Thus, although theoretically the smaller stomata in the rain-fed tobacco plants would lead to decreased maximum conductance, their reduced size may help to protect productivity, conserving water under stress conditions and prioritizing CO_2_ uptake under favorable conditions.

For many of the traits related to biomass productivity, consistent significant differences (2018: *P*<0.05; 2020: *P*<0.05) were seen between treatments between both field seasons (Fig. 2). This coincides with previous literature which documents that drought stress or water limitation in plants can result in reduced total dry weight, total leaf area, and specific leaf area (Poorter *et al.* 2012; Mishra and Panda, 2017; Wellstein *et al.* 2017; Panda *et al.* 2021). Total leaf area decreases under water stress conditions as the plant invests less in leaves and stems to reduce evaporative area for water loss (Eziz *et al.* 2017). Reduced total leaf area can then result in lower total dry weight as the amount of radiation intercepted to drive photosynthesis is diminished (Jamieson *et al.* 1995). Additionally, lower SLA is associated with increased water use efficiency under water stress (Wellstein *et al.* 2017). Data from these two field seasons suggest that the rain-fed plants were deploying strategies to conserve limited water in the field (Wellstein *et al.* 2017).

However, why might this water stress be insufficient to consistently affect photosynthetic traits? The results indicate that the irrigation treatment was insufficient to cause significant differences in the rate of carboxylation or electron transport between the treatments (Supplementary Fig. S7). *V*_cmax_ and maximum rate of electron transport (*J*_max_) tend to decrease under water limitation, resulting in an overall lowered *A*_n_ (Flexas *et al.* 2004; Vaz *et al.* 2010; Rho *et al.* 2012). However, these parameters also can recover quickly from water limitation after the onset of a precipitation event (Vaz *et al.* 2010). Additionally, in some cases it has been shown that *V*_cmax_ can be unaffected by water limitation, unless the plants were adapted to wet conditions prior (Cano *et al.* 2013). As such, it is possible that either *V*_cmax_ in rain-fed plants recovered from water stress during a precipitation event before the measurement of the *A*_n_*–C*_i_ curve values or that the capacity of RuBP carboxylation was unaffected by the treatment throughout the field season.

In conclusion, our results demonstrate that guard-cell-targeted overexpression of *AtHXK1* may have the potential to generate plants with more conservative water use throughout the growing season under field conditions and moderate water limitation, without a significant yield penalty. In farmers’ fields, the resulting conservation of soil water content may reduce the impact of late-season dry spells and decrease the need for water supplementation via irrigation.

## Supplementary data

The following supplementary data are available at *JXB* online.

Fig. S1. AtHXK1 expression levels.

Fig. S2. Soil water content from probe readings.

Fig. S3. Leaf count.

Fig. S4. Seedling dry weight and leaf count 2018.

Fig. S5. Seedling dry weight and leaf count 2020.

Fig. S6. Leaf area ratio.

Fig. S7. CO_2_ response curves of net assimilation rate.

Fig. S8. Stomatal density and anatomy.

Fig. S9. Flowering time.

Table S1. Primer sequences.

Table S2. Group means and Tukey’s HSD groupings for *A*_n_.

Table S3. Group means and Tukey’s HSD groupings for *g*_s_.

Table S4. Group means and Tukey’s HSD groupings for *C*_i_.

Table S5. Group means and Tukey’s HSD groupings for iWUE.

erac218_suppl_Supplementary_Material

## Data Availability

All data supporting the findings of this study are available within the paper and within its supplementary materials published online.

## Abbreviations

*A*_n_: net CO_2_ assimilation
*C*_i_: intercellular CO_2_ concentration
*g*_s_: stomatal conductance of water
iWUE: intrinsic water use efficiency (*A*_n_/*g*_s_)
TPU: triosephosphate utilization
*V*_cmax_: maximum rate of carboxylation
