# Supplementary material for: Guard-cell-targeted overexpression of Arabidopsis Hexokinase 1 can improve water use efficiency in field-grown tobacco plants
Source: J Exp Bot. 2022 May 20;73(16):5745–57. doi: 10.1093/jxb/erac218 (PMC9467653; doi:10.1093/jxb/erac218)
Supplement: erac218_suppl_Supplementary_Material [file erac218_suppl_supplementary_material.pdf]

SUPPLEMENTAL MATERIALS

Guard-cell Targeted Overexpression of Arabidopsis Hexokinase 1 May Improve Water Use Efficiency in Field-Grown Tobacco Plants

TABLES:

Supplementary Table 1. Primer sequences used for PCR verification of T-DNA integration and RT-qPCR determination of AtHXK1 and NtActin expression levels.

Gene/T-DNA	Forward primer (5'-3')	Reverse primer (5'-3')	Locus
AtHXK1	AAACCTACCCAAAGAGCGCC	TGACGCCTTAGAACTTGGCT	AT4G29130
NtActin	CCTGAGGTCCTTTTCCAACCA	GATTCCGGCAGCTTCCATT	XM_016658252
35S::AtHXK1	CCGACAGTGGTCCCAAAGAT	CAGTTTCGAGATCGGAGTCG	n.a.

Supplementary Table 2. Mean discrimination of *A* using Tukey's Honest Significant Difference (HSD) on dates where a significant difference was detected between genotypes for *A*.

Date	Treatment	Means (µmol m^{-2} s^{-1})	Group
June 15, 2018	Irrigated	16.18	A
	Rainfed	12.07	B
June 30, 2018	Irrigated	27.64	A
	Rainfed	21.35	B
July 8, 2020	Irrigated	22.58	A
	Rainfed	12.26	B
July 23, 2020	Rainfed	26.6	A
	Irrigated	25.2	B

Supplementary Table 3. Mean discrimination of g_s using Tukey's Honest Significant Difference (HSD) on dates where a significant difference was detected between genotypes for g_s.

Date	Genotype	Means (mol m^{-2} s^{-1})	Group
June 30, 2018	WT – Irrigated	0.656	A
	35SHXK2, 35SHXK5 - Irrigated	0.649, 0.602	AB
	GCHXK2 – Irrigated	0.578	B
	WT - Rainfed	0.327	C
	35SHXK2, 35SHXK5 – Rainfed	0.320, 0.272	CD
	GCHXK2 - Rainfed	0.249	D
July 8, 2020	Irrigated	0.655	A
	Rainfed	0.165	B
July 29, 2020	WT	0.991	A
	35SHXK2	0.911	AB
	GCHXK2	0.852	BC
	35SHXK5	0.786	C

Supplementary Table 4. Mean discrimination of C_i using Tukey's Honest Significant Difference (HSD) on dates where a significant difference was detected between genotypes for C_i.

Date	Genotype	Mean (μmol mol^{-1})	Group
June 30, 2018	WT – Irrigated	297	A
	35SHXK2, 35SHXK5 – Irrigated	294, 291	AB
	GCHXK2 – Irrigated	282	B
	WT – Rainfed	241	C
	35SHXK2, 35SHXK5 – Rainfed	239, 236	CD
	GCHXK2 - Rainfed	226	D
July 8, 2020	35SHXK5 – Irrigated	328	A
	35SHXK2, GCHXK2 – Irrigated	312, 307	AB
	WT – Irrigated	306	B
	35SHXK5 – Rainfed	266	C
	35SHXK2, GCHXK2 – Rainfed	249, 245	CD
	WT - Rainfed	243	D
July 29, 2020	WT	337	A
	35SHXK2, GCHXK2,	332, 329	AB
	35SHXK5	329	B

Supplementary Table 5. Mean discrimination of intrinsic water-use efficiency (*iWUE* = A_n/g_s) using Tukey's Honest Significant Difference (HSD) on dates where a significant difference was detected between genotypes for *iWUE*.

**Date**	**Genotype**	**Means** (µmol CO_2 mol H_2O^{-1})	**Group**
iWUE- June 30, 2018	H2, WT, H5, GC – Irrigated	46.02, 47.55, 48,51, 49.98	A
	WT, H2 – Rainfed	75.87, 82.12	B
	H5 – Rainfed	84.08	BC
	GC – Rainfed	95.34	C
iWUE – July 8, 2020	H5, GC, WT, H2 - Irrigated	40.54, 44, 29, 45.29, 47.43	A
	H5 – Rainfed	70.03	B
	H2 – Rainfed	82.1	BC
	GC, WT – Rainfed	91.37, 92.53	C
iWUE – July 29, 2020	WT	29.52	A
	H2	32.1	AB
	GC, H5	33.97, 34.4	B

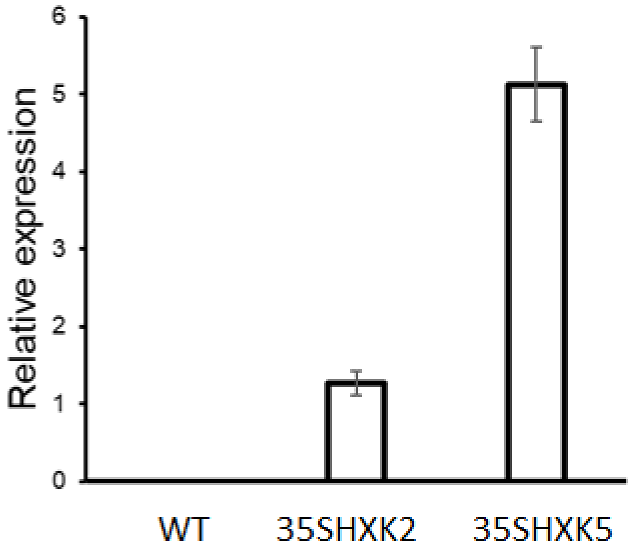

Supplementary Figure 1. Relative expression level of AtHXK1 in Tobacco 35SHXK lines. Relative expression of AtHXK1 was examined using RNA extracted from young fully developed leaves of wild-type (WT) and two transgenic lines, 35SHXK2 and 35SHXK5. The tobacco Actin gene (XM_016658252) was used for normalization of expression. Error bars show standard error, n = 6.

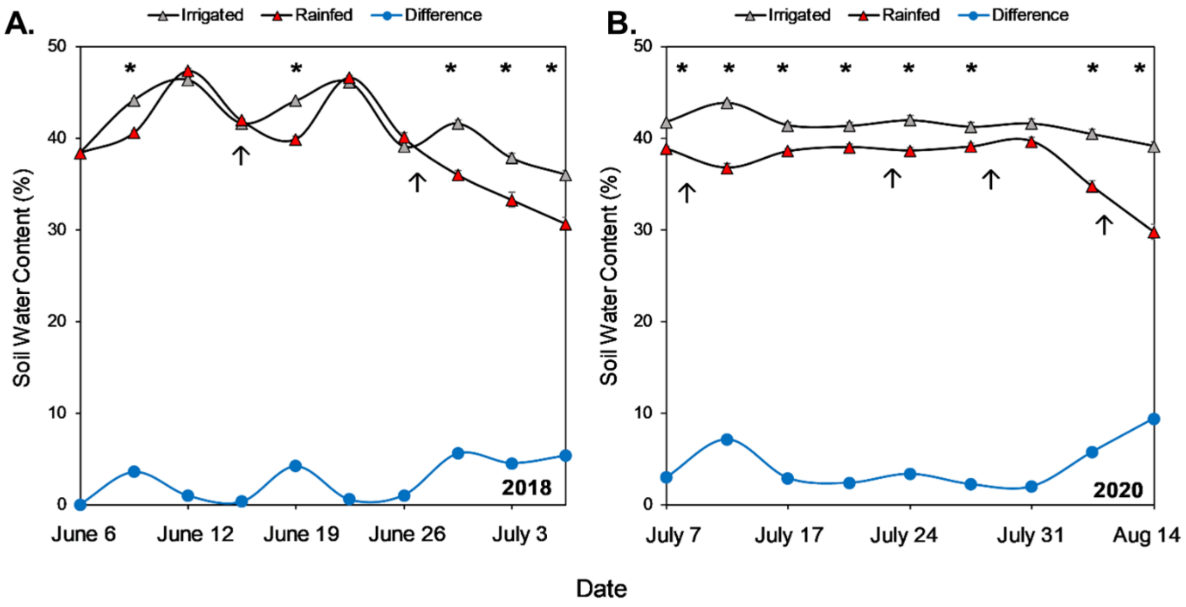

Supplementary Figure 2. Volumetric water content of top 15 cm layer of soil profile, measured at mid-day by inserting a handheld probe in both 2018 and 2020 field seasons. Open symbols show individual measurements in irrigated (grey) and rain-fed (red) plots and filled symbols connected with solid lines show mean values for irrigated (grey) or rain-fed (red) plots and the difference between means (blue). Asterisks indicate significant differences between irrigated and rain fed soil moisture content ($p < 0.05$). The arrows are indicative of when diurnal gas exchange measurements were taken in the field.

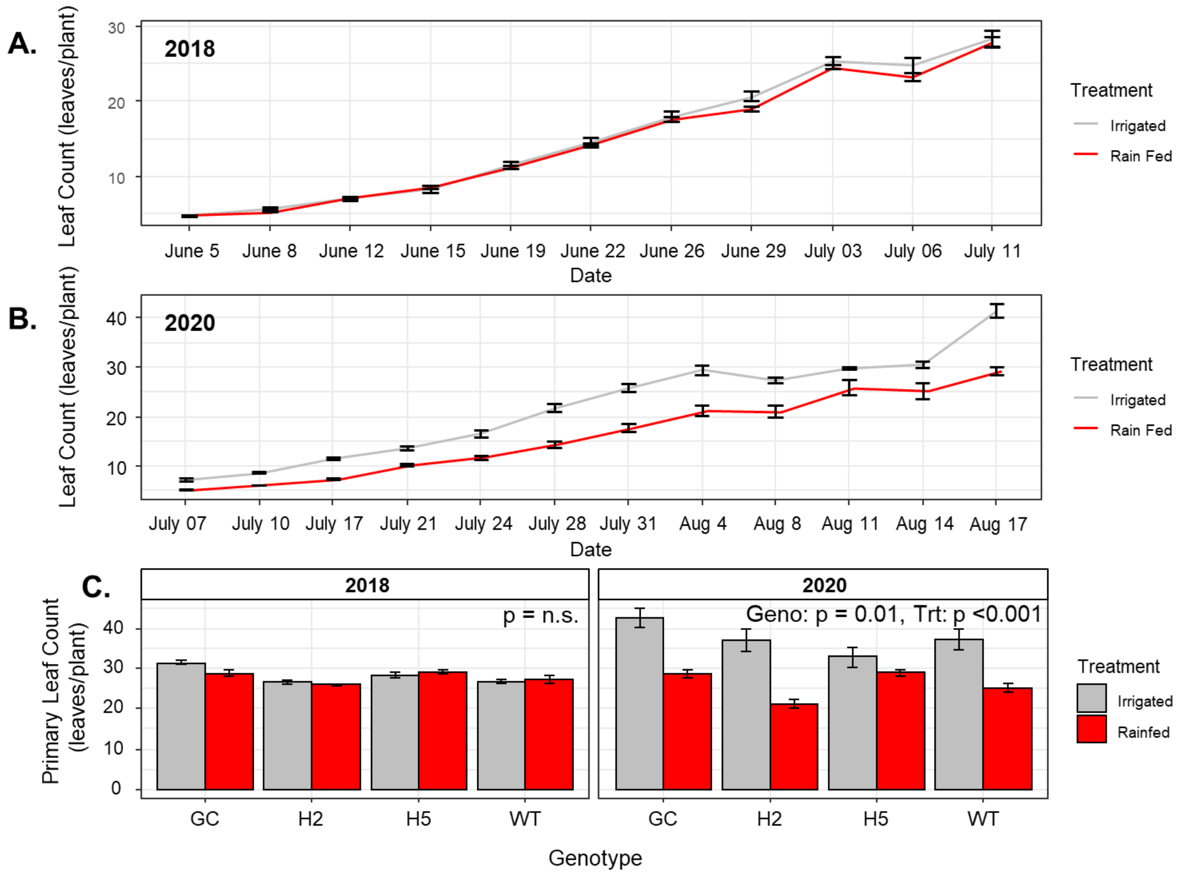

Supplementary Figure 3. Leaf counts throughout the 2018 and 2020 field seasons. **A.** Leaf emergence as quantified by leaf counts throughout the 2018 field season, where July 11, 2018, marks the day the experiment was harvested. **B.** Leaf emergence as quantified by leaf counts throughout the 2020 field season, where August 17, 2020, marks the day the experiment was harvested. **C.** Final leaf counts as determined during harvest in 2018 and 2020, respectively. In A and B Error bars show standard error, n = 24. In C error bars show standard error, while n = 6 for each genotype in each treatment. Abbreviations of genotypes are as follow: GCHXK2 (GC), 35SHXK2 (H2), 35SHXK5 (H5), wild type (WT).

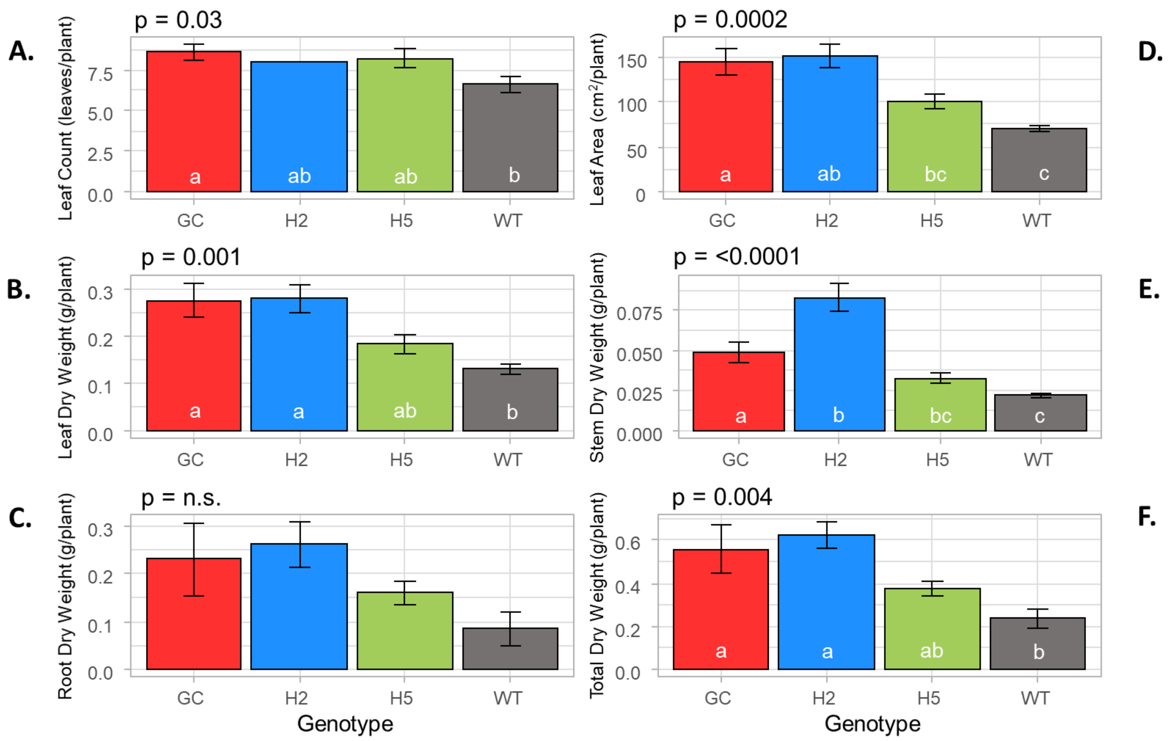

Supplementary Figure 4. Initial biomass and leaf counts for transgenic tobacco plants overexpressing AtHXK1 either constitutively (35SHXK2 and 35SHXK5) or in guard cells (GCHXK2) and corresponding wild-type (WT-control) during the 2018 field season. Abbreviations of genotypes are as follow: GCHXK2 (GC), 35SHXK2 (H2), 35SHXK5 (H5), wild type (WT).

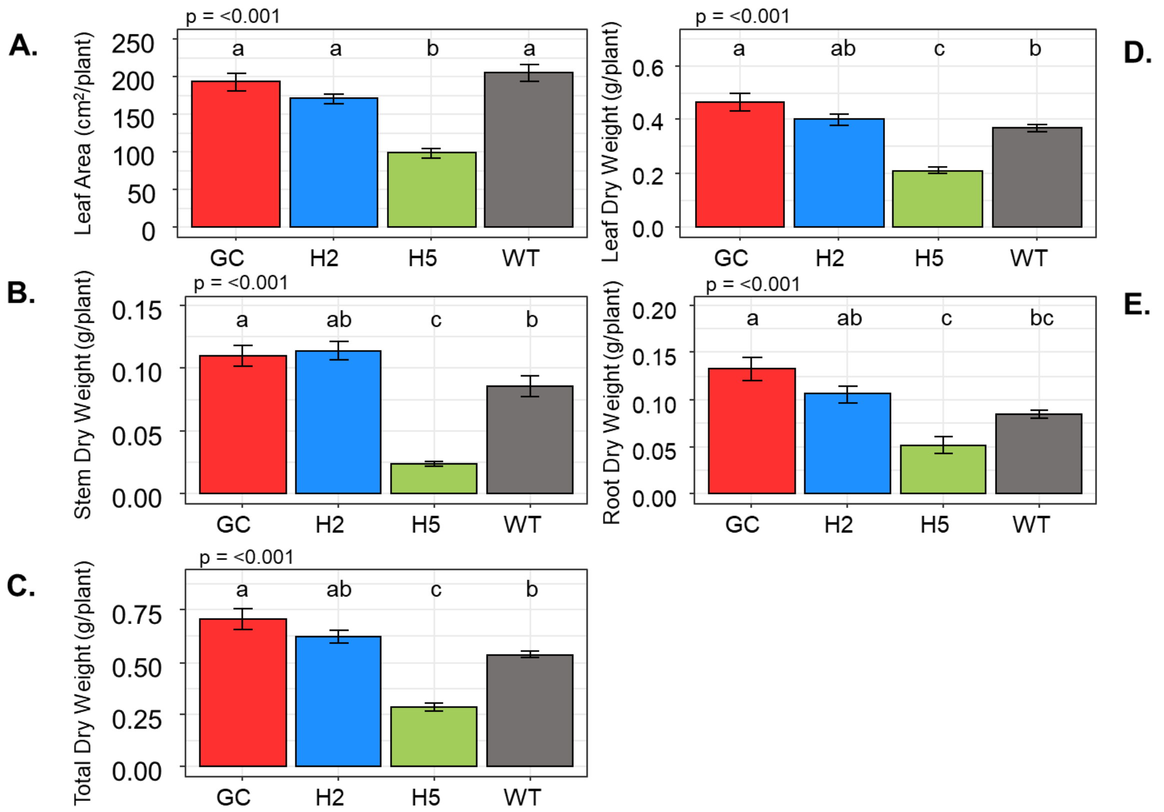

Supplementary Figure 5. Initial biomass for transgenic tobacco plants overexpressing AtHXK1 either constitutively (35SHXK2 and 35SHXK5) or in guard cells (GCHXK2) and corresponding wild-type (WT-control) during the 2020 field season. Abbreviations of genotypes are as follow: GCHXK2 (GC), 35SHXK2 (H2), 35SHXK5 (H5), wild type (WT).

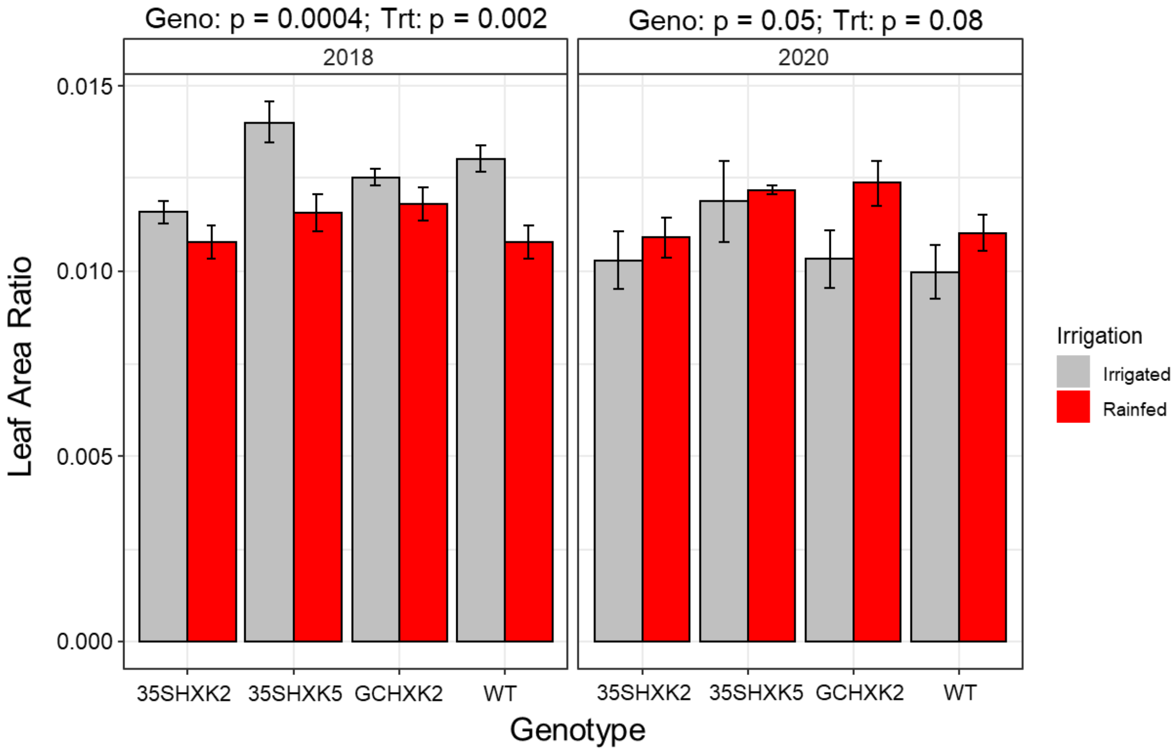

Supplementary Figure 6. Biomass allocation pattern to leaves of field-grown transgenic tobacco plants overexpressing either constitutively (35SHXK2 and 35SHXK5) or in guard cells (GCHXK2) and corresponding wild-type (WT- control). n = 6 per genotype and treatment.

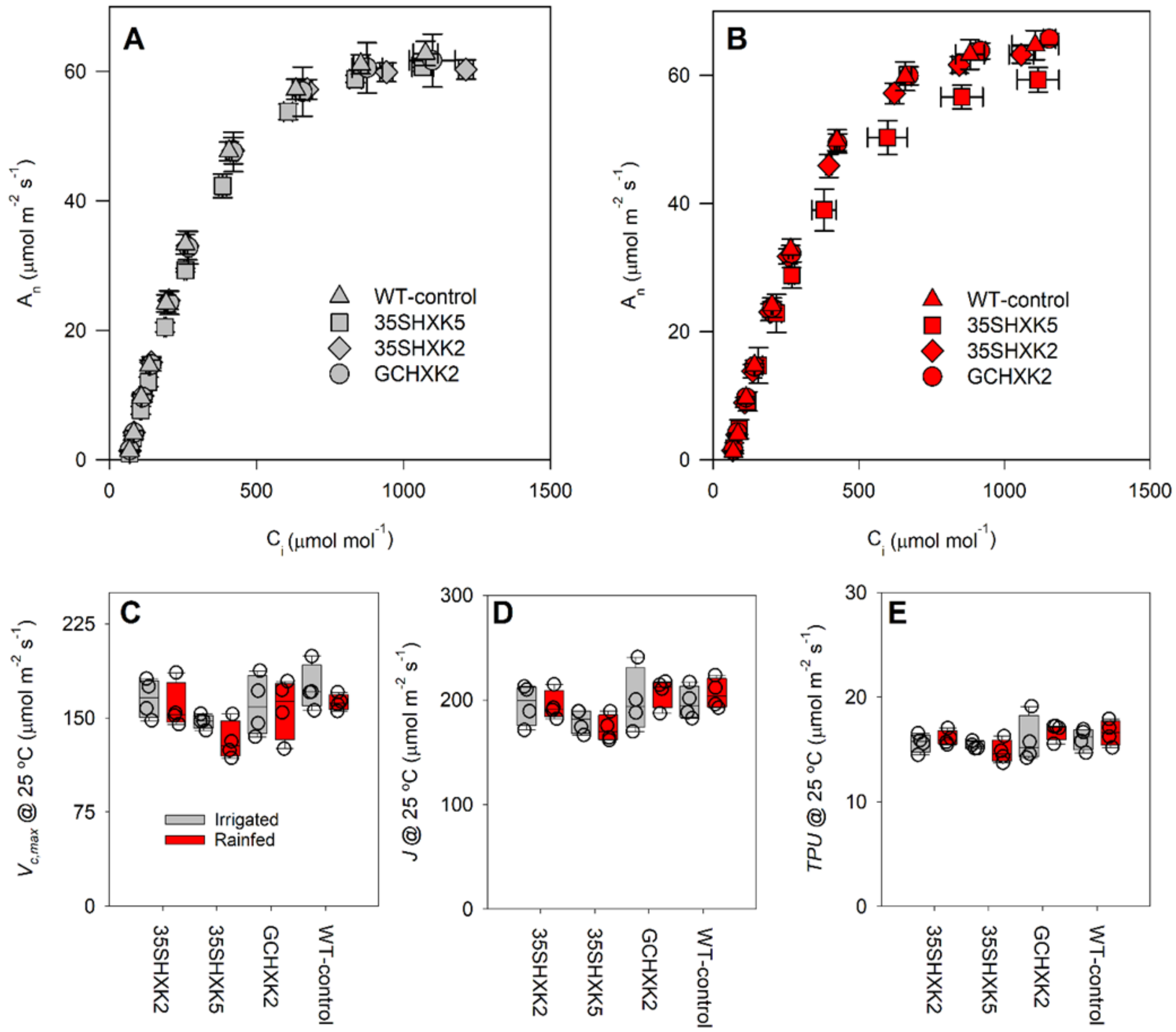

Supplementary Figure 7. Net CO_2 assimilation rate (A_n) as a function of intercellular CO_2 concentration (C_i) in field-grown transgenic tobacco plants overexpressing AtHXK1 either constitutively (35SHXK2 and 35SHXK5) or in guard cells (GCHXK2) and corresponding wild-type (WT-control). **(A)** A_n/C_i responses in irrigated plots; **(B)** A_n/C_i responses in rain-fed plots; **(C)** Maximum rate of ribulose bisphosphate carboxylation per genotype / irrigation combination, derived from data in panel (A) and (B), standardized to 25 °C; **(D)** Rate of ribulose bisphosphate regeneration at 2000 µmol m^{-2} s^{-1} per genotype / irrigation combination, derived from data in (A) and (B), standardized to 25 °C; **(E)** Triose phosphate utilization capacity per genotype / irrigation combination derived from data in (A) and (B), standardized to 25 °C. Open symbols in C-E show parameter estimates from individual response curves. Box plots show central tendency and distributions of observations for irrigated (gray) and rain-fed (red) plants (n = 4).

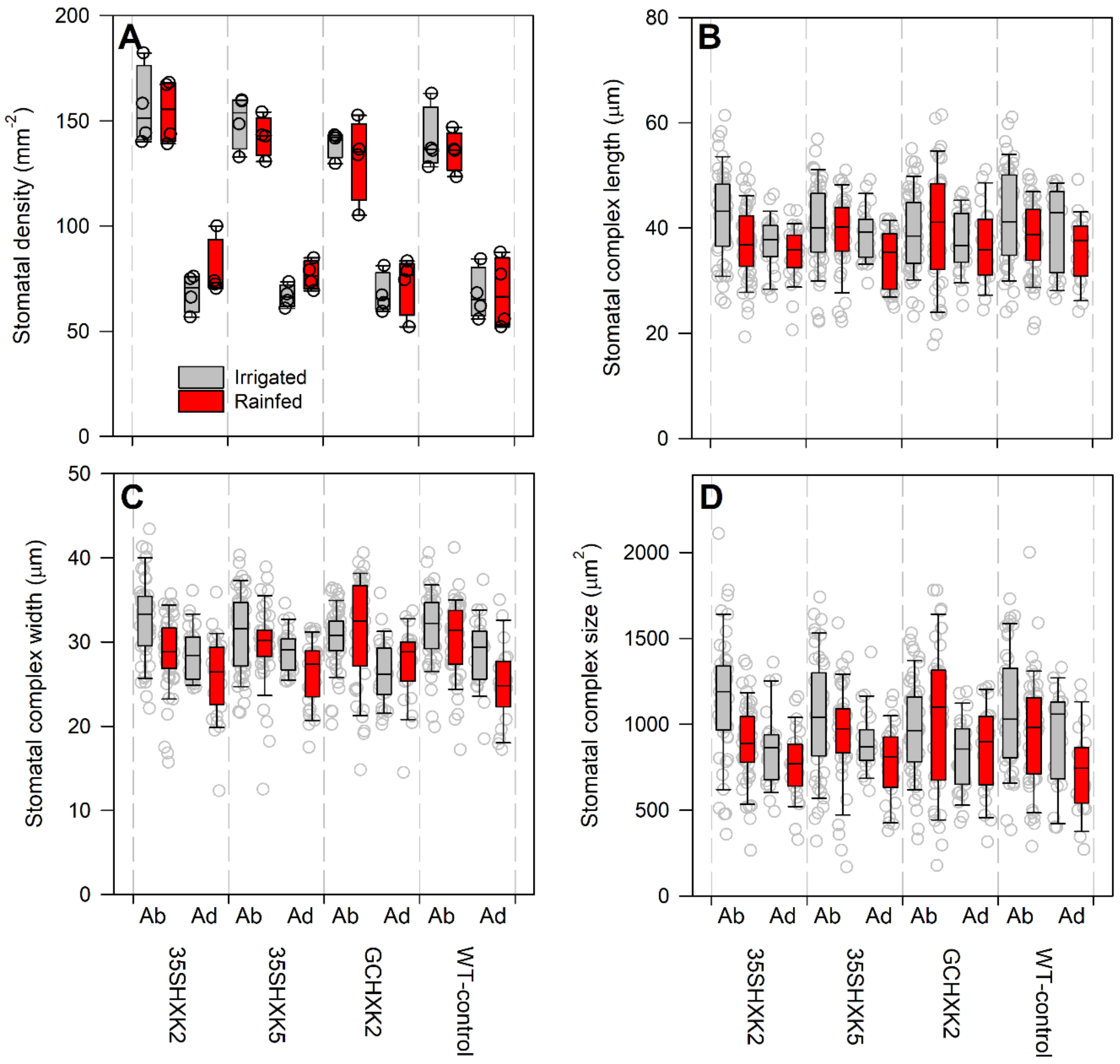

Supplementary Figure 8. Stomatal density and anatomy in field-grown transgenic tobacco plants overexpressing AtHXK1 either constitutively (35SHXK2 and 35SHXK5) or in guard cells (GCHXK2) and corresponding wild-type (WT-control). **(A)** Stomatal density on adaxial (Ad) and abaxial (Ab) leaf sides, determined using optical topometry on three technical replicates and four biological replicates. Open symbols show means per biological replicate. Box plots show central tendency and distribution of biological replicates of irrigated (gray) and rain-fed (red) plants. **(B)** Length of stomatal complex; **(C)** width of stomatal complex; **(D)** Area of stomatal complex. Open symbols in B-D show individual measurements per stomatal complex, box plots show central tendency and distribution of stomatal properties in irrigated (gray) and rain-fed (red) plants.

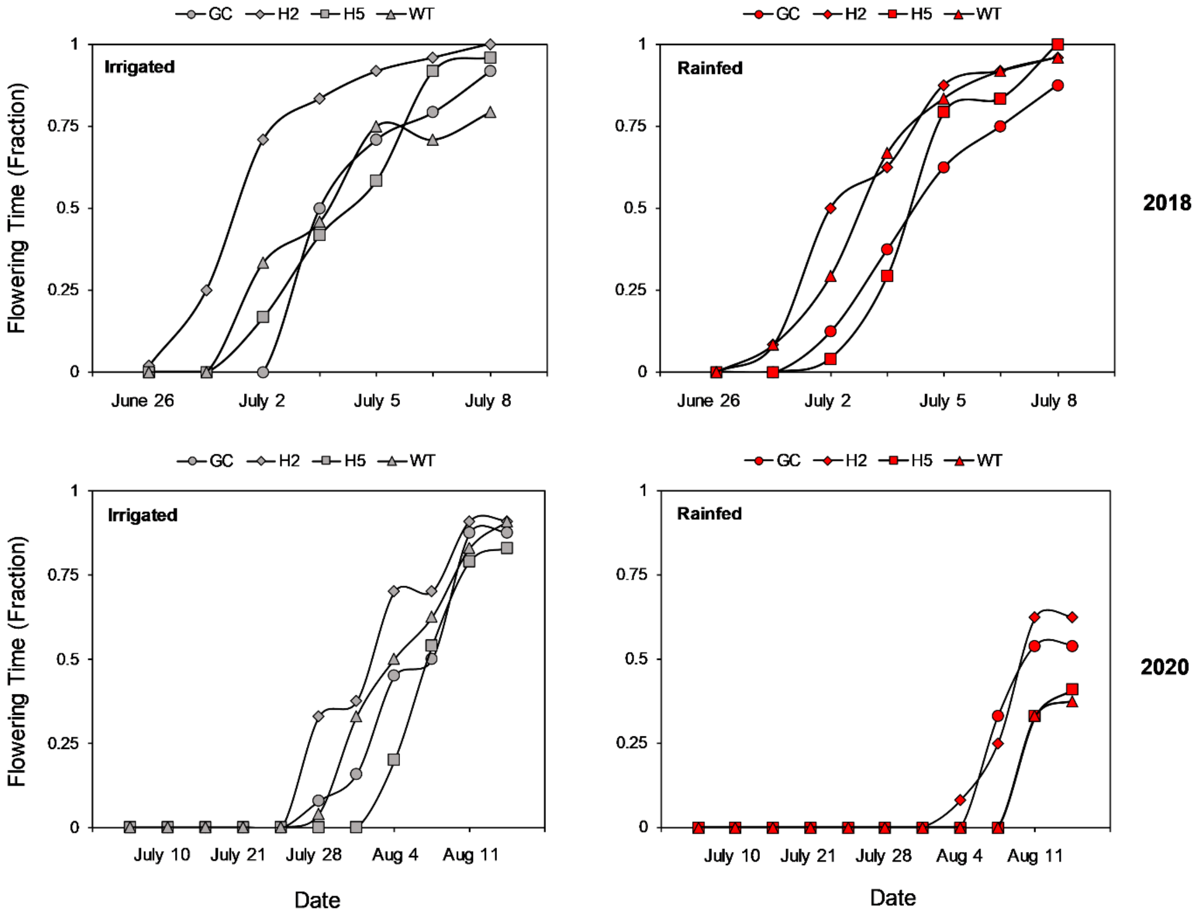

Supplementary Figure 9. Flowering time for transgenic tobacco plants overexpressing AtHXK1 either constitutively (35SHXK2 and 35SHXK5) or in guard cells (GCHXK2) and corresponding wild-type (WT-control) during the 2018 and 2020 field seasons.
